# Supplementary material for: Microwave excitation of atomic scale superconducting bound states
Source: Nat Commun. 2023 Oct 25;14:6794. doi: 10.1038/s41467-023-42454-5 (PMC10600199; doi:10.1038/s41467-023-42454-5)
Supplement: Supplementary file 1 — Supplementary Information [file 41467_2023_42454_MOESM1_ESM.pdf]

Supplementary Information for "Microwave Excitation of Atomic Scale Superconducting Bound States"

Janis Siebrecht,[1] Haonan Huang,[1] Piotr Kot,[1] Robert Drost,[1] Ciprian Padurariu,[2]
Björn Kubala,[2,3] Joachim Ankerhold,[2] Juan Carlos Cuevas,[4] and Christian R. Ast[1,*]

[1]*Max-Planck-Institut für Festkörperforschung, Heisenbergstraße 1, 70569 Stuttgart, Germany*
[2]*Institut für Komplexe Quantensysteme and IQST, Universität Ulm, Albert-Einstein-Allee 11, 89069 Ulm, Germany*
[3]*Institute of Quantum Technologies, German Aerospace Center (DLR), Söflinger Straße 100, 89077, Ulm, Germany*
[4]*Departamento de Física Teórica de la Materia Condensada and Condensed Matter Physics Center (IFIMAC), Universidad Autónoma de Madrid, 28049 Madrid, Spain*
(Dated: October 9, 2023)

SUPPLEMENTARY NOTE 1: METHODS

Tip and Sample Preparation

The V(100) sample was cleaned by repeated Argon ion bombardment and annealing to 700 °C. The typical appearance of the surface are square terraces with an oxygen reconstruction as shown in Fig. S1 The tip was made superconducting using field emission (40 V bias voltage and 15 μA current). By controlled dipping (4 nm dip at 100 mV), YSR states were created on the apex of the tip [1, 2]. The exact composition of the tip apex is not known simply by the way tips are prepared in general, but a likely scenario is that the YSR impurity on the tip is made of oxygen or carbon atom(s) picked up from the surface such that it leaves a free spin to interact with the superconductor. We purposefully chose to use YSR tips for our experiment as it generally gave better stability of the junction. We believe that the impurity is spin-½, which has already been discussed previously leading to an overall consistent picture [1–5].

For the present system, depending on the exact composition of the apex, the YSR states appear at different energies, allowing us to tune the YSR energy to the relevant frequency range between 60-100 GHz.

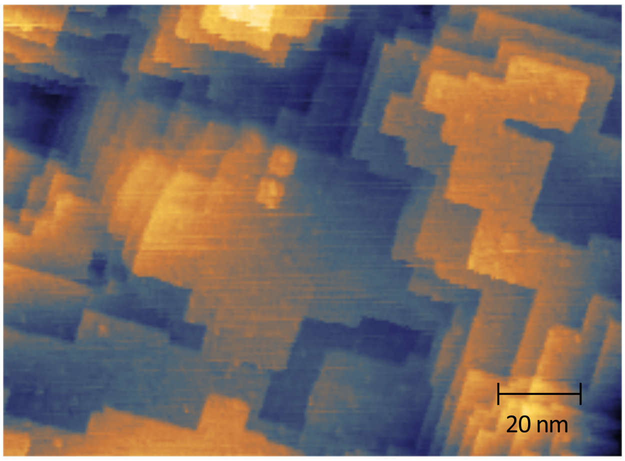

Figure S1: **Topography of the V(100) surface.** The data was obtained at a set point of 100 pA with a bias voltage of 3 mV.

Microwave Transmission

The microwave setup is similar to the one introduced in Ref. [6]. The microwave source is a Keysight 8257D frequency generator (up to 20 GHz), whose frequency output is multiplied by a factor of six using a Virginia Diodes WR12SGX device. A millimeter wave 511E attenuator is used to tune the attenuation. In the vacuum chamber, we use semirigid Cu coaxial cables, which, starting at the 4 K stage, is replaced by a superconducting semirigid coaxial cable. Finally, the radiation is transmitted through vacuum to the tunnel junction using a custom made bow-tie antenna on a chip [6]. To measure the transfer function, we use a feedback scheme as shown in Fig. S2. We broaden the peak by applying a lock-in amplitude and then reduce the attenuation until the peak drops below a threshold of 80 % of its maximum value. Then the ratio of the actual peak height A_ω to the original peak height A_0 is used to calculated the ac amplitude according to: $\frac{A_\omega}{A_0} = J_0^2\left(\frac{eV_{\mathrm{ac}}}{\hbar\omega}\right)$, where J_0 is the zeroth order Bessel function of the first kind.

SUPPLEMENTARY NOTE 2: FULL GREEN'S FUNCTION THEORY

General theory

In this section we show how the general theory of photon-assisted tunneling in superconducting junctions developed in Ref. 7 can be adapted to the description of the microwave-assisted tunneling through a magnetic impurity coupled to superconducting leads. Our goal is to calculate the current through a voltage biased superconducting tunnel junction in the presence of a monochromatic radiation of frequency ω_{r}. For simplicity, we focus on the case of a single channel contact. We assume that the external radiation produces an effective time-dependent voltage $V(t) = V + V_{\mathrm{ac}} \sin \omega_{\mathrm{r}} t$. The task now is to extend the theory for multiple Andreev reflections (MARs) to the case of such a time-dependent voltage, for which the so-called Hamiltonian approach is a convenient starting point [8]. The irradiated single channel superconducting tunnel junction can be described by means of the fol-

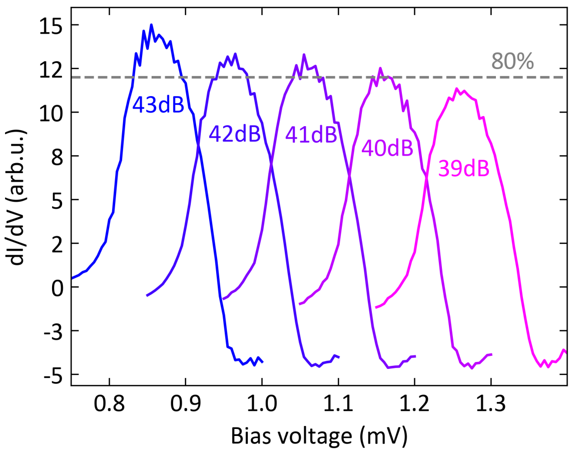

Figure S2: **Illustration of the algorithm for the transfer function determination.** The plot shows differential conductance spectra of a coherence peak at different values of microwave attenuation. The spectra are offset in voltage for clarity. Starting from a previously determined attenuation, the attenuation is reduced in steps of 1 dB until the peak height is below the threshold of 80% of the original peak. The data was measured at a frequency of 61.1 GHz, and a setpoint current of 100 pA at a bias voltage of 3 mV.

lowing tight-binding-like Hamiltonian [8]

$$\hat{H} = \hat{H}_{\rm L} + \hat{H}_{\rm R} + \sum_\sigma \left\{ t\, c^\dagger_{{\rm L}\sigma} c_{{\rm R}\sigma} + t^*\, c^\dagger_{{\rm R}\sigma} c_{{\rm L}\sigma} \right\}, \qquad \text{(S1)}$$

where $H_{\rm L,R}$ are the Hamiltonians following Bardeen-Cooper-Schrieffer (BCS) theory for the isolated electrodes. In the coupling term, L and R stand for the outermost sites of each electrode, and t is a hopping parameter describing the coupling between these sites. This parameter determines the normal state transmission coefficient τ of this model in a way that depends on the nature of the tunnel junction. For instance, in a tunnel junction formed by two conventional BCS superconductors, the transmission adopts the form

$$\tau = \frac{4(t/W)^2}{[1+(t/W)^2]^2}, \text{ where } W = 1/\pi\rho_F, \qquad \text{(S2)}$$

with ρ_F being the electrodes' density of states at the Fermi level [8].

In this model the current evaluated at the interface between the two electrodes adopts the form

$$I(t) = \frac{ie}{\hbar} \sum_\sigma \left\{ t\langle c^\dagger_{{\rm L}\sigma}(t) c_{{\rm R}\sigma}(t)\rangle - t^* \langle c^\dagger_{{\rm R}\sigma}(t) c_{{\rm L}\sigma}(t)\rangle \right\}. \qquad \text{(S3)}$$

The nonequilibrium expectation values in Eq. (S3) can be expressed in terms of the Keldysh-Green functions $\hat{G}^{+-}_{ij}$ (i,j = L,R), which in the 2×2 Nambu representation read

$$\hat{G}^{+-}_{ij}(t,t') = i \begin{pmatrix} \langle c^\dagger_{j\uparrow}(t') c_{i\uparrow}(t)\rangle & \langle c_{j\downarrow}(t') c_{i\uparrow}(t)\rangle \\ \langle c^\dagger_{j\uparrow}(t') c^\dagger_{i\downarrow}(t)\rangle & \langle c_{j\downarrow}(t') c^\dagger_{i\downarrow}(t)\rangle \end{pmatrix}. \qquad \text{(S4)}$$

Thus, the current can be now written as

$$I(t) = \frac{e}{\hbar} \,{\rm Tr}\left[\hat{\tau}_3 \left(\hat{t}_{\rm LR}(t) \hat{G}^{+-}_{\rm RL}(t,t) - \hat{G}^{+-}_{\rm LR}(t,t)\hat{t}_{\rm RL}(t) \right)\right], \qquad \text{(S5)}$$

where $\hat{\tau}_3$ is the corresponding Pauli matrix in Nambu space, Tr denotes the trace in Nambu space and the $\hat{t}$'s are given by

$$\hat{t}_{\rm LR}(t) = \hat{t}^\dagger_{\rm RL}(t) = \begin{pmatrix} t e^{i\phi(t)/2} & 0 \\ 0 & -t^* e^{-i\phi(t)/2} \end{pmatrix}. \qquad \text{(S6)}$$

Here, $\phi(t) = \phi_0 + \omega_0 t + 2\alpha \cos\omega_{\rm r} t$ is the time-dependent superconducting phase difference. In this expression, ϕ_0 is the dc part of the superconducting phase difference, $\omega_0 = 2eV/\hbar$ is the Josephson frequency, and the constant $\alpha = eV_{\rm ac}/(\hbar\omega_{\rm r})$ measures the strength of the coupling to the electromagnetic field, and is proportional to the square root of the radiation power.

Using the relation

$$e^{iz\cos\phi} = \sum_k i^k J_k(z) e^{ik\phi}, \qquad \text{(S7)}$$

where $J_k(z)$ is the Bessel function of order k, one can write the time dependence of the hopping as follows

$$t e^{i\phi(t)/2} = t e^{i(\phi_0+\omega_0 t)/2} \sum_k i^m J_k(\alpha) e^{ik\omega_{\rm r} t}. \qquad \text{(S8)}$$

In order to determine the Green functions we follow a perturbative scheme and treat the coupling term in the Hamiltonian (cf. Eq. (S1)) as a perturbation. The unperturbed Green functions $\hat{g}^{r,a}$ correspond to the uncoupled electrodes in equilibrium, where the superscript r, a denotes the retarded and advanced components, respectively. Following Ref. [8], one can express the current in terms of a T-matrix, rather than in terms of the Green functions. The T-matrix associated to the time-dependent perturbation of Eq. (S6) is defined as

$$\hat{T}^{r,a} = \hat{t} + \hat{t} \circ \hat{g}^{r,a} \circ \hat{T}^{r,a}, \qquad \text{(S9)}$$

where the $\circ$ product is a shorthand for integration over intermediate time arguments. With this definition, it is easy to show that

$$\hat{T}^{r,a}_{\rm LR} = \hat{t}_{\rm LR} + \hat{t}_{\rm LR} \circ \hat{g}^{r,a}_{\rm R} \circ \hat{t}_{\rm RL} \circ \hat{g}^{r,a}_{\rm L} \circ \hat{T}^{r,a}_{\rm LR}, \qquad \text{(S10)}$$
$$\hat{T}^{r,a}_{\rm RL} = \hat{t}_{\rm RL} + \hat{t}_{\rm RL} \circ \hat{g}^{r,a}_{\rm L} \circ \hat{t}_{\rm LR} \circ \hat{g}^{r,a}_{\rm R} \circ \hat{T}^{r,a}_{\rm RL}. \qquad \text{(S11)}$$

As shown in Ref. [8], the current in terms of the T-matrix components reads

$$I(t) = \frac{e}{\hbar}{\rm Tr}\left[\hat{\tau}_3 \left(\hat{T}^r_{\rm LR} \circ \hat{g}^{+-}_{\rm R} \circ \hat{T}^a_{\rm RL} \circ \hat{g}^a_{\rm L} - \hat{g}^r_{\rm L} \circ \hat{T}^r_{\rm LR} \circ \hat{g}^{+-}_{\rm R} \circ \hat{T}^a_{\rm RL} + \hat{g}^r_{\rm R} \circ \hat{T}^r_{\rm RL} \circ \hat{g}^{+-}_{\rm L} \circ \hat{T}^a_{\rm LR} - \hat{T}^r_{\rm RL} \circ \hat{g}^{+-}_{\rm L} \circ \hat{T}^a_{\rm LR} \circ \hat{g}^a_{\rm R} \right)\right]. \qquad \text{(S12)}$$

In order to solve the T-matrix integral equation, it is convenient to Fourier transform with respect to the temporal arguments:

$$\hat{T}(t,t') = \frac{1}{2\pi}\int dE \int dE'\, e^{-iEt/\hbar} e^{iE't'/\hbar}\, \hat{T}(E,E'). \qquad \text{(S13)}$$

Due to time dependence of the coupling element (see Eq. (S6)), one can show that $\hat{T}(E,E')$ admits the following solution:

$$\hat{T}(E,E') = \sum_{n,m} \hat{T}(E, E + neV + m\hbar\omega_{\mathrm{r}})\delta(E - E' + neV + m\hbar\omega_{\mathrm{r}}). \tag{S14}$$

Thus, one can finally write down the current as

$$I(t) = \sum_{n,m} I_n^m \exp\left[i\left(n\phi_0 + n\omega_0 t + m\omega_{\mathrm{r}} t\right)\right], \tag{S15}$$

where the current amplitudes I_n^m can be expressed in terms of the T-matrix Fourier components, $\hat{T}^{kl}_{nm}(E) \equiv \hat{T}(E + neV + k\hbar\omega_{\mathrm{r}}, E + meV + l\hbar\omega_{\mathrm{r}})$, in the following way

$$I_n^m = \frac{e}{h}\int dE \sum_{i,k} \mathrm{Tr}\left[\hat{\tau}_3\left(\overset{r}{\hat{T}}{}^{0k}_{\mathrm{LR},0i}\overset{+-}{\hat{g}}{}^{k}_{\mathrm{R},i}\overset{a}{\hat{T}}{}^{km}_{\mathrm{RL},in}\overset{a}{\hat{g}}{}^{m}_{\mathrm{L},n} - \overset{r}{\hat{g}}{}^{0}_{\mathrm{L},0}\overset{r}{\hat{T}}{}^{0k}_{\mathrm{LR},0i}\overset{+-}{\hat{g}}{}^{k}_{\mathrm{R},i}\overset{a}{\hat{T}}{}^{km}_{\mathrm{RL},in} + \overset{r}{\hat{g}}{}^{0}_{\mathrm{R},0}\overset{r}{\hat{T}}{}^{0k}_{\mathrm{RL},0i}\overset{+-}{\hat{g}}{}^{k}_{\mathrm{L},i}\overset{a}{\hat{T}}{}^{km}_{\mathrm{LR},in} - \overset{r}{\hat{T}}{}^{0k}_{\mathrm{RL},0i}\overset{+-}{\hat{g}}{}^{k}_{\mathrm{L},i}\overset{a}{\hat{T}}{}^{km}_{\mathrm{LR},in}\overset{a}{\hat{g}}{}^{m}_{\mathrm{R},n}\right)\right]. \tag{S16}$$

We are interested in the dc current I_{dc}. In general, this current is the sum of two contributions $I_{\mathrm{dc}} = I_{\mathrm{B}} + I_{\mathrm{Shapiro}}$, where $I_{\mathrm{B}} \equiv I_0^0$ (cf. Eq. (S15)) is a background current and $I_{\mathrm{Shapiro}} = \sum_{n,m} I_n^m e^{in\phi_0}\delta(V - V_n^m)$ is the current from Shapiro steps contribution at discrete voltages $V_n^m = (m/n)\hbar\omega_{\mathrm{r}}/2e$. We shall ignore the contribution from the Shapiro steps in the following and focus only on the background current.

Using the relations

$$(\overset{a}{\hat{T}}{}^{kl}_{\mathrm{RL},ij})^\dagger = \overset{r}{\hat{T}}{}^{lk}_{\mathrm{LR},ji} \quad \text{and} \quad \overset{r}{\hat{T}}{}^{kl}_{\mathrm{LR},ij} = (-1)^{k-l}\overset{a}{\hat{T}}{}^{kl}_{\mathrm{LR},ij}, \tag{S17}$$

which can be demonstrated using the corresponding T-matrix equations for these components, we can write the dc current exclusively in terms of $\hat{T}_i^k \equiv \overset{a}{\hat{T}}{}^{k0}_{\mathrm{LR},i0}$ as follows

$$I_{\mathrm{dc}} = \frac{2e}{h}\int dE \sum_{i,k} \mathrm{ReTr}\left[\hat{\tau}_3\left(\overset{a}{\hat{g}}{}^{k}_{\mathrm{L},i}\hat{T}_i^k\overset{+-}{\hat{g}}{}^{0}_{\mathrm{R},0}\hat{T}_i^{k\dagger} - \hat{T}_i^{k\dagger}\overset{+-}{\hat{g}}{}^{k}_{\mathrm{L},i}\hat{T}_i^k\overset{a}{\hat{g}}{}^{0}_{\mathrm{R},0}\right)\right]. \tag{S18}$$

Finally, the $\hat{T}_i^k$ fulfill the following set of linear algebraic equations

$$\hat{T}_i^k = \hat{t}_i^k + \sum_l \left\{\hat{\mathcal{E}}^{kl}_{i,i}\hat{T}_i^l + \hat{\mathcal{V}}^{kl}_{i,i+2}\hat{T}^l_{i+2} + \hat{\mathcal{V}}^{kl}_{i,i-2}\hat{T}^l_{i-2}\right\}, \tag{S19}$$

where the different matrix coefficients adopt the following form in terms of the unperturbed Green functions

$$\begin{aligned}
\hat{t}_i^k &= \frac{t}{2}J_k(\alpha)\left[i^k(\hat{1} + \hat{\tau}_3)\,\delta_{i,-1} - (-i)^k(\hat{1} - \hat{\tau}_3)\,\delta_{i,1}\right] \\
\hat{\mathcal{E}}^{kl}_{i,i} &= t^2 i^{k+l}\sum_j (-1)^j J_{k-j}(\alpha)J_{j-l}(\alpha)\begin{pmatrix} (g^j_{R,i+1})_{11}(g^l_{L,i})_{11} & (g^j_{R,i+1})_{11}(g^l_{L,i})_{12} \\ (g^j_{R,i-1})_{22}(g^l_{L,i})_{21} & (g^j_{R,i-1})_{22}(g^l_{L,i})_{22} \end{pmatrix} \\
\hat{\mathcal{V}}^{kl}_{i,i+2} &= -t^2 i^{k-l}\sum_j J_{k-j}(\alpha)J_{j-l}(\alpha)(g^j_{R,i+1})_{12}\begin{pmatrix} (g^l_{L,i+2})_{21} & (g^l_{L,i+2})_{22} \\ 0 & 0 \end{pmatrix} \\
\hat{\mathcal{V}}^{kl}_{i,i-2} &= -t^2 i^{l-k}\sum_j J_{k-j}(\alpha)J_{j-l}(\alpha)(g^j_{R,i-1})_{21}\begin{pmatrix} 0 & 0 \\ (g^l_{L,i-2})_{11} & (g^l_{L,i-2})_{12} \end{pmatrix},
\end{aligned}$$

where we have used the shorthand notation $(g^k_{\mathrm{L},i})_{\alpha,\beta} = g^a_{\mathrm{L},\alpha,\beta}(E + ieV + k\hbar\omega_{\mathrm{r}})$, where $\alpha,\beta = 1,2$ are indexes in Nambu space.

Approximations

In general, one has to solve Eq. (S19) numerically to then evaluate the current via Eq. (S18). However, in low-transmission junctions there are a number of approximations that one can make. In the deep tunnel regime (when the tunnel coupling is the smallest energy scale), one can use the following approximation for the solution of Eq. (S19):

$$\hat{T}_i^k \approx \hat{t}_i^k \quad (i = \pm 1). \tag{S20}$$

This leads to the standard Tien-Gordon result for the tunneling of single quasiparticles (see below).

If we want to consider at least the lowest order Andreev reflection, the next approximation is

$$\begin{aligned} \hat{T}^k_1 &\approx \hat{t}^k_1 + \sum_l \hat{\mathcal{V}}^{kl}_{1,-1} \hat{t}^l_{-1}, \\ \hat{T}^k_{-1} &\approx \hat{t}^k_{-1} + \sum_l \hat{\mathcal{V}}^{kl}_{-1,1} \hat{t}^l_1. \end{aligned} \tag{S21}$$

Using this approximation in Eq. (S18), we get the lowest-order approximation for the contributions of both the quasiparticle current ($|t|^2$) and the Andreev reflection ($|t|^4$). Additionally, one gets terms like a higher order contribution for the quasiparticle current.

The previous two approximations are perturbative in nature and may lead to divergencies, if they are not properly regularized. This is what happens, for instance, when there is a bound state inside the gap with a very long lifetime. In those cases, one can fix that problem by solving the following closed system for $\hat{T}^k_1$ and $\hat{T}^k_{-1}$:

$$\begin{aligned} \hat{T}^k_1 &= \hat{t}^k_i + \sum_l \left\{ \hat{\mathcal{E}}^{kl}_{1,1} \hat{T}^l_1 + \hat{\mathcal{V}}^{kl}_{1,-1} \hat{T}^l_{-1} \right\} \\ \hat{T}^k_{-1} &= \hat{t}^k_{-1} + \sum_l \left\{ \hat{\mathcal{E}}^{kl}_{-1,-1} \hat{T}^l_1 + \hat{\mathcal{V}}^{kl}_{-1,1} \hat{T}^l_1 \right\}, \end{aligned} \tag{S22}$$

whose solution is

$$\begin{aligned} \hat{T}_1 &= \left[1 - \hat{\mathcal{E}}_{1,1} - \hat{\mathcal{V}}_{1,-1} \left[1 - \hat{\mathcal{E}}_{-1,-1} \right]^{-1} \hat{\mathcal{V}}_{-1,1} \right]^{-1} \left(\hat{t}_1 + \hat{\mathcal{V}}_{1,-1} \left[1 - \hat{\mathcal{E}}_{-1,-1} \right]^{-1} \hat{t}_{-1} \right), \\ \hat{T}_{-1} &= \left[1 - \hat{\mathcal{E}}_{-1,-1} - \hat{\mathcal{V}}_{-1,1} \left[1 - \hat{\mathcal{E}}_{1,1} \right]^{-1} \hat{\mathcal{V}}_{1,-1} \right]^{-1} \left(\hat{t}_{-1} + \hat{\mathcal{V}}_{-1,1} \left[1 - \hat{\mathcal{E}}_{1,1} \right]^{-1} \hat{t}_1 \right). \end{aligned} \tag{S23}$$

Note that in Eq. (S23) the different matrices have to be understood as big matrices in microwave space. It is worth remarking that this approximation exactly reproduces the results for the YSR problem for the typical transmissions of the experiments.

Actually, there are intermediate approximations that seem to work very well. For instance, to regularize the quasiparticle term the following approximation suffices

$$\begin{aligned} \hat{T}_1 &\approx \left[1 - \hat{\mathcal{E}}_{1,1} \right]^{-1} \hat{t}_1, \\ \hat{T}_{-1} &\approx \left[1 - \hat{\mathcal{E}}_{-1,-1} \right]^{-1} \hat{t}_{-1}, \end{aligned} \tag{S24}$$

Here, one can ignore the off-diagonal elements (in Nambu space) of $\hat{\mathcal{E}}_{i,i}$.

The minimal approximation to regularize the Andreev term is given by

$$\begin{aligned} \hat{T}_1 &\approx \left[1 - \hat{\mathcal{E}}_{1,1} \right]^{-1} \left(\hat{t}_1 + \hat{\mathcal{V}}_{1,-1} \left[1 - \hat{\mathcal{E}}_{-1,-1} \right]^{-1} \hat{t}_{-1} \right), \\ \hat{T}_{-1} &\approx \left[1 - \hat{\mathcal{E}}_{-1,-1} \right]^{-1} \left(\hat{t}_{-1} + \hat{\mathcal{V}}_{-1,1} \left[1 - \hat{\mathcal{E}}_{1,1} \right]^{-1} \hat{t}_1 \right) \end{aligned} \tag{S25}$$

where again one can ignore the off-diagonal elements (in Nambu space) of $\hat{\mathcal{E}}_{i,i}$.

YSR states + microwaves

To describe the tunneling through an YSR impurity we use the mean-field Anderson impurity model put forward in Refs. [1, 9]. Within this model the Green's functions of the electrodes are given as follows. For the left electrode, which is superconducting, we use the standard BCS Green's functions:

$$\hat{g}_{\mathrm{L}}(E) = \frac{-\pi N_{0,\mathrm{L}}}{\sqrt{\Delta_{\mathrm{L}}^2 - E^2}} \left[E\tau_0 + \Delta_{\mathrm{L}}\tau_1 \right], \tag{S26}$$

where $N_{0,\mathrm{L}}$ is the density of states at the Fermi energy of the left electrode in the normal conducting state. On the other hand, the Green functions for the right electrode features a superconducting electrode with the impurity, adopt the form [9]

$$\hat{g}_{\mathrm{R}}(E) = \frac{1}{D(E)} \begin{pmatrix} E\Gamma_{\mathrm{R}} + (E + U - J)\sqrt{\Delta_{\mathrm{R}}^2 - E^2} & \Gamma_{\mathrm{R}}\Delta_{\mathrm{R}} \\ \Gamma_{\mathrm{R}}\Delta_{\mathrm{R}} & E\Gamma_{\mathrm{R}} + (E - U - J)\sqrt{\Delta_{\mathrm{R}}^2 - E^2} \end{pmatrix}, \tag{S27}$$

where

$$D(E) = 2\Gamma_{\mathrm{R}} E(E - J) + \left[(E - J)^2 - U^2 - \Gamma_{\mathrm{R}}^2 \right] \sqrt{\Delta_{\mathrm{R}}^2 - E^2}. \tag{S28}$$

Here, we have defined the tunneling rate $\Gamma_L = \pi N_{0,L} t_L^2$ (a similar rate $\Gamma_R = \pi N_{0,R} t_R^2$ describes the strength of the tip-impurity coupling).

Let us recall that the condition for the appearance of superconducting bound states is $D(E) = 0$. In particular, the spin-induced YSR states appear in the limit $|J| \gg \Delta_R$ (and they are inside the gap when also $\Gamma_R \gg \Delta_R$). In this case, there is a pair of fully spin-polarized YSR bound states at energies $\pm\epsilon$, where

$$\epsilon = \Delta_R \frac{J^2 - \Gamma_R^2 - U^2}{\sqrt{\left[\Gamma_R^2 + (J-U)^2\right]\left[\Gamma_R^2 + (J+U)^2\right]}}, \tag{S29}$$

which in the electron-hole symmetric case $U = 0$ reduces to

$$\epsilon = \Delta_R \frac{J^2 - \Gamma_R^2}{J^2 + \Gamma_R^2}. \tag{S30}$$

In this case, using the approximation of Eq. (S20) we arrive at the following expression for the quasiparticle current to the lowest order in the tunnel coupling:

$$I_{qp} \approx \frac{4e\pi^2|t|^2}{h} \sum_k J_k^2(\alpha) \int_{-\infty}^{\infty} \rho_L(E - eV + k\hbar\omega_r)\rho_R(E)\left[f(E - eV + \hbar\omega_r) - f(E)\right] dE, \tag{S31}$$

where $\rho_i(E)$ is the density of states of electrode i and $f(E)$ is the Fermi function. This is simply the standard Tien-Gordon result. Thus, because of the presence of YSR states inside the gap (with energy $\epsilon > 0$), one expects the microwaves to give rise to a series of conductance peaks at $eV = \Delta_S + \epsilon + m\hbar\omega_r$ with a height that should evolve with the microwave power as $J_m^2(\alpha)$.

Using the approximation of Eq. (S21) and selecting the contribution to the resonant Andreev reflection, we arrive at the following expression for the current due to the resonant Andreev reflection (to lowest order in the tunnel coupling):

$$I_{AR} \approx \frac{8e\pi^2|t|^4}{h} \sum_{k,l} J_k^2(\alpha) J_l^2(\alpha) \int_{-\infty}^{\infty} \rho_L(E - eV + k\hbar\omega_r)\, \rho_L(E + eV + l\hbar\omega_r)\, |(g_R)_{12}(E)|^2 \times \left[f(E - eV + k\hbar\omega_r) - f(E + eV + l\hbar\omega_r)\right] dE, \tag{S32}$$

where $(g_R)_{12}(E)$ is the anomalous Green function at the impurity site and it is given in Eq. (S27).

Equations (S31) and (S32) nicely explain the physics of the experimental observation. The last remaining thing is to establish what are the simplest expressions that regularize these equations when the YSR states are very long lived. After some careful analysis, we have arrived at the following regularized expressions:

$$I_{qp}^{(reg)} \approx \frac{4e\pi^2|t|^2}{h} \sum_k J_k^2(\alpha) \int_{-\infty}^{\infty} \left\{ \frac{\rho_L(E - eV + k\hbar\omega_r)\rho_{R,1}(E)}{\left|1 - |t|^2 J_k^2(\alpha) (\overset{a}{g}{}^k_{L,-1})_{11} (\overset{a}{g}{}^0_{R,0})_{11}\right|^2} \left[f(E - eV + \hbar\omega_r) - f(E)\right] - \frac{\rho_L(E + eV + k\hbar\omega_r)\rho_{R,2}(E)}{\left|1 - |t|^2 J_k^2(\alpha) (\overset{a}{g}{}^k_{L,1})_{22} (\overset{a}{g}{}^0_{R,0})_{22}\right|^2} \left[f(E + eV + \hbar\omega_r) - f(E)\right] \right\} dE, \tag{S33}$$

where $\rho_{R,i}(E) = (1/\pi)\mathrm{Im}\left\{(\overset{a}{g}{}^0_{R,0})_{ii}\right\}$ $(i = 1, 2)$, $[\rho_R(E) = \rho_{R,1}(E) + \rho_{R,2}(-E)]$, and

$$I_{AR}^{(reg)} \approx \frac{8e\pi^2|t|^4}{h} \sum_{k,l} J_k^2(\alpha) J_l^2(\alpha) \int_{-\infty}^{\infty} \frac{\rho_L(E - eV + k\hbar\omega_r)\, \rho_L(E + eV + l\hbar\omega_r)\, |(g_R)_{12}(E)|^2}{\left|\left[1 - |t|^2 J_k^2(\alpha) (\overset{a}{g}{}^k_{L,-1})_{11} (\overset{a}{g}{}^0_{R,0})_{11}\right]\left[1 - |t|^2 J_l^2(\alpha) (\overset{a}{g}{}^l_{L,1})_{22} (\overset{a}{g}{}^0_{R,0})_{22}\right]\right|^2} \times \left[f(E - eV + k\hbar\omega_r) - f(E + eV + l\hbar\omega_r)\right] dE. \tag{S34}$$

Notice that the only difference with respect to the perturbative results above is the presence of a denominator

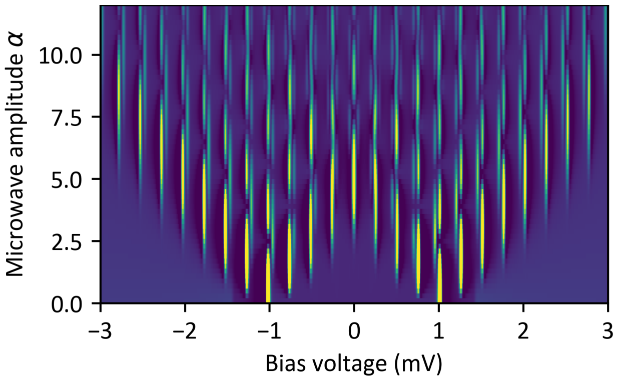

Figure S3: **Calculated differential conductance map as function of microwave amplitude** Calculation of the YSR state replica as function of bias voltage and microwave amplitude using the regularized quasiparticle and Andreev currents (Eq. (S33) and (S34)). The excited state tunneling is clearly visible for higher amplitudes.

that regularizes the eventual divergencies. For a comparison of these approximations with the full Green's function model, see Section .

In Fig. S3, we show an example of the differential conductance as a function of the bias voltage V and α computed with Eqs. (S33) and (S34), i.e. we computed the total current as the sum of those two contributions. The parameters are given in Table SI. As one can see, these equations reproduce all the salient features of the experiment.

Finally, to make contact with recent results [10], we define the energy-dependent tunneling rates for electrons Γ_e and holes Γ_h as

$$\Gamma_e(E) = 2\pi|t|^2\rho_L(E)\tilde{u}^2, \quad (S35)$$
$$\Gamma_h(E) = 2\pi|t|^2\rho_L(E)\tilde{v}^2, \quad (S36)$$

where the coherent factors $\tilde{u}^2$ and $\tilde{v}^2$ in our model are given by

$$\tilde{u}^2 = \frac{2\Delta_R J\Gamma_R^2}{\left[\Gamma_R^2 + (J+U)^2\right]} \frac{1}{\sqrt{\left[\Gamma_R^2 + (J+U)^2\right]\left[\Gamma_R^2 + (J-U)^2\right]}},$$
$$\tilde{v}^2 = \frac{2\Delta_R J\Gamma_R^2}{\left[\Gamma_R^2 + (J-U)^2\right]} \frac{1}{\sqrt{\left[\Gamma_R^2 + (J+U)^2\right]\left[\Gamma_R^2 + (J-U)^2\right]}}.$$

With these definitions, the anomalous Green's function in the impurity can be approximated by (for energies close to the YSR energy)

$$|(g_R)_{12}(E)|^2 \approx \frac{\tilde{u}^2\tilde{v}^2}{(E-\epsilon)^2 + \eta_R^2}. \quad (S37)$$

Thus, the perturbative expression for the current contribution of the resonant AR of Eq. (S32) becomes

$$I_{AR} \approx \frac{2e}{h}\sum_{k,l} J_k^2(\alpha)J_l^2(\alpha) \times \int_{-\infty}^{\infty} \frac{\Gamma_e(E - eV - k\hbar\omega_r)\,\Gamma_h(E + eV + l\hbar\omega_r)}{(E-\epsilon)^2 + \eta_R^2}\left[f(E - eV - k\hbar\omega_r) - f(E + eV + l\hbar\omega_r)\right] dE. \quad (S38)$$

This expression has to be compared with Eq. (40) in Ref. [10]. A list of parameters that were used to obtain the calculated spectra in the different figures is given in Table SI.

SUPPLEMENTARY NOTE 3: VALIDITY OF THE MODELS

Resonant tunneling easily involves higher order tunneling long before higher orders become significant in nonresonant tunneling. This is particularly the case when resonant tunneling is combined with the interaction with microwaves. We, therefore, evaluate up to which transparencies such approximations are valid in different models. Here, we compare three models:

1. Full Green's functions model (Eq. (S18) with Eq. (S19), black lines in Figures S4 and S5)
2. Green's function model to first order in Andreev reflections (Eq. (S18) with Eq. (S23), blue lines in Figures S4 and S5)
3. Regularized Andreev model (Eq. (S33) and (S34), red lines in Figures S4 and S5)

To assess the agreement of model A referenced to model B with functions $f_A(x)$ and $f_B(x)$, we evaluate the mean squared deviation referenced to the mean squared deviation from zero:

$$\chi^2 = \frac{\int (f_A(x) - f_B(x))^2\,dx}{\int f_B(x)^2 dx} \quad (S39)$$

We plot the evolution of the spectrum without microwaves as a function of conductance for each model in Fig. S4(a). Every spectrum is normalized by the normal state tunneling conductance. For each spectrum, we calculated the deviation referenced to the full Green's function model and plot this in Fig. S4(b). The regularized Andreev model deviates by more than 5% from the full calculation at a transparency of 4×10^{-2}, whereas the deviations of the first order model only become relevant at a transparency of 1×10^{-1}.

In contrast to that, when the microwaves are included, the three models become inconsistent much faster. Figure S5(a) shows four spectra for each model calculated at different conductances. At the highest conductance, the reg-

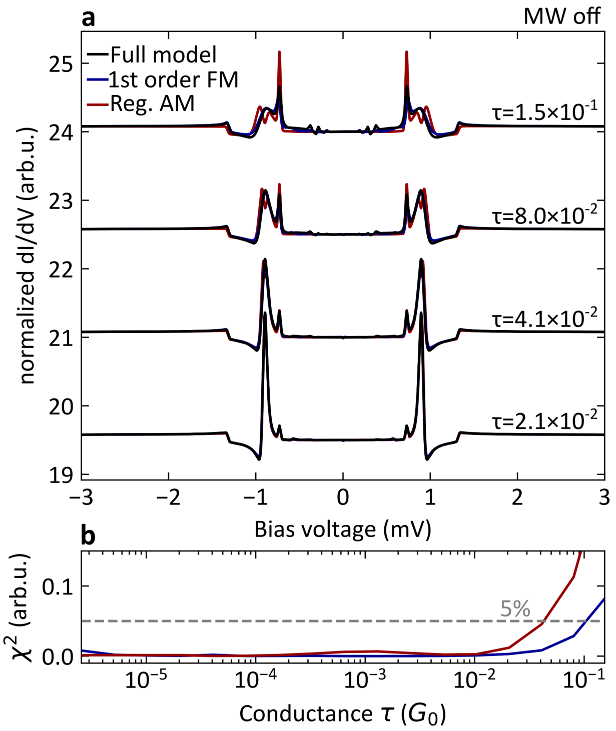

Figure S4: **Comparison of the different models without microwaves.** (a) Differential conductance spectra calculated using the different models as function of selected junction transparencies τ with the microwaves turned off. The full Green's function model is labeled "Full model", the Green's function model is labeled "1st order FM", and the regularized Andreev model is labeled "Reg. AM". The regularized Andreev model is calculated from the sum of the quasiparticle current and the Andreev current. (b) Deviations of the approximations from the full Green's function model as function of junction transparency τ. Both approximations only fail for high transparencies when higher order processes become relevant also for nonresonant tunneling processes.

ularized Andreev model shows deviations in peak height, amplitude and even peak position. The first order approximation still performs much better. This means that the interference of higher order processes is crucial to properly describe the spectrum under microwave irradiation. In Fig. S5(b), the deviations start about two orders of magnitude sooner for the regularized Andreev model, which crosses the 5% mark at a transparency of 5×10^{-4}. The first order approximation crosses the 5% mark at a transparency of 8×10^{-2}.

The comparison of the behaviour with and without microwaves leads to an important conclusion for the experimental data. Firstly, for measurements without microwaves, the three models remain consistent up to about $\tau = 2 \times 10^{-2}$, which corresponds to roughly 12 nA for a set point bias voltage of 4 mV. This means that for the lifetime broadening of 0.6 µeV (cf. Table SI) used here and for typical setpoint currents of $O(100\,\mathrm{pA})$, higher order contributions are not relevant. If the lifetime broadening is smaller, higher order contributions will become relevant at lower transparencies (i.e. smaller setpoint currents) [1, 11]. In contrast to that, the regularized Andreev

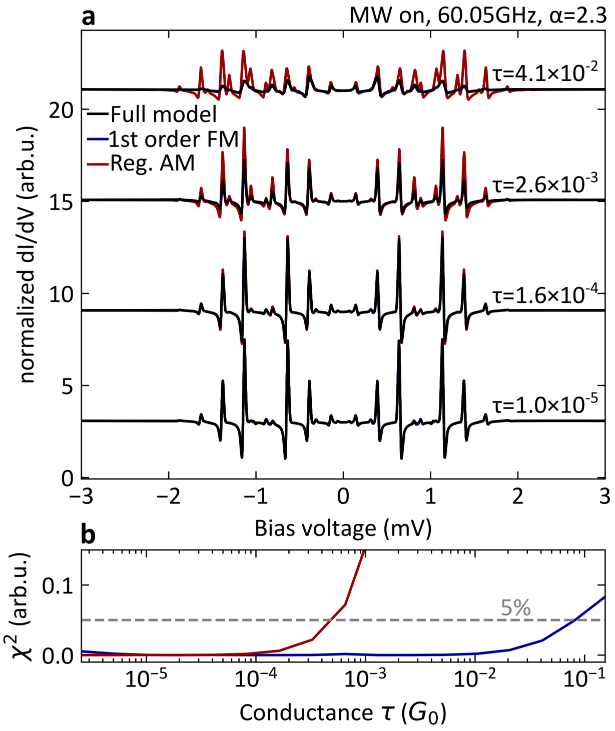

Figure S5: **Comparison of the different models with microwaves.** (a) Differential conductance spectra calculated using the different models as function of selected junction transparencies τ with the microwaves turned on. The full Green's function model is labeled "Full model", the Green's function model is labeled "1st order FM", and the regularized Andreev model is labeled "Reg. AM". The regularized Andreev model is calculated from the sum of the quasiparticle current and the Andreev current. (b) Deviations of the approximations from the full Green's function model as function of junction transparency τ. The first order model fails at somewhat lower transparencies than without microwaves, but the regularized model fails for two orders of magnitude lower transparencies than before. This indicates that interactions/interference between the resonant processes and the microwaves cannot be neglected for a quantitative and even a qualitative agreement.

model with microwaves shows significant disagreement already at a transparency of 5×10^{-4}, corresponding to about 150 pA. This means that for typical measurements with microwaves and YSR states a full Green's function approach is necessary. The resonances due to the interactions with the microwave lead to a failure of the lowest order approximation [10]. A list of parameters that were used to obtain the calculated spectra in the different figures (both main text and supplementary information) is given in Table SI.

We also mention that in Fig. 1(c) of the main text, there are some peaks between −1.2 mV and −0.7 mV as well as between 0.7 mV and 1.2 mV, which are not captured by the model. These peaks are replicas of the small coherence peak at $\pm\Delta$, which arise from a finite filling of the gap (Dynes parameter). They are not due to Andreev reflections because the junction transmission is too low.

Figure	Δ_L	Δ_R	η_L	η_R	J	U	Γ_L	$\hbar\omega_r$	α
1b	0.73	0.59	0.1	0.1	75.5	0	0.07	0	0
1c	0.73	0.59	0.1	0.1	75.5	0	0.07	0.248	3.5
2c	0.74	0.69	0.1	0.1	68.0	25	0.04	0.252	~
3b	0.73	0.69	0.1	0.1	64.5	25	0.04	~	3
S3	0.73	0.69	0.1	0.1	64.5	25	0.04	0.252	~
S4	0.73	0.59	0.1	0.6	75.3	0	~	0	0
S5	0.73	0.59	0.1	0.6	75.3	0	~	0.248	2.3

Table SI: **Fitting Parameters** Table of fit parameters that were used to calculate the spectra in the corresponding figures. The parameters are given in meV, except for α, which is dimensionless, and $\eta_{L,R}$, which is measured in μeV. The temperature was set to be 0.56 K, the coupling of the impurity to the tip $\Gamma_R = 100$ meV, and an overall Gaussian broadening was chosen to be 12.5 μeV. The right electrode (R) carries the YSR state, while the left electrode (L) features an empty gap. For the fit in Fig. 1(b), the channel transmissions are $\tau_{YSR} = 140\,\text{nS} = 1.8 \times 10^{-3}\,G_0$ and $\tau_{BCS} = 30\,\text{nS} = 3.9 \times 10^{-4}\,G_0$ for the BCS and the YSR channel, respectively.

SUPPLEMENTARY NOTE 4: DERIVATION OF THE SIMPLIFIED GROUND STATE AND EXCITED STATE TUNNELING MODEL

In the following, we will derive a simplified model to highlight the roles of the replicas in step ① and step ③ of ground state and excited state tunneling (cf. schematic in Fig. 1(e) and (g) of the main text). We simplify the tunneling and focus on the interplay of the Bessel functions and exchange of energy quanta. We start with Eq. (S38). In the case of long-lived YSR states, i.e. very small η_S, we can approximate the Lorentzian of the YSR state by a Dirac delta-function $\frac{1}{(E-\epsilon)^2+\eta_R^2} \approx \frac{\pi}{\eta_R}\delta(E-\epsilon)$. This step solves the integral in Eq. (S38) and the Andreev current becomes

$$I_{AR} \approx \frac{e}{\hbar\eta_R}\sum_{k,l} J_k^2(\alpha)J_l^2(\alpha)\Gamma_e(\epsilon - eV - k\hbar\omega_r)\,\Gamma_h(\epsilon + eV + l\hbar\omega_r) \times [f(\epsilon - eV - k\hbar\omega_r) - f(\epsilon + eV + l\hbar\omega_r)] \quad \text{(S40)}$$

Each tunneling rate $\Gamma_{e,h}$ has two peaks at $\pm\Delta$, such that we have a total of four peaks in the spectrum. To separate out these peaks, we use the Heaviside step function $\theta(E)$ to define $\Gamma^{\pm}_{e,h}(E) = \theta(\pm E)\Gamma_{e,h}(E)$ so that we can split $\Gamma_{e,h}(E)$ into

$$\Gamma_{e,h}(E) = \Gamma^{+}_{e,h}(E) + \Gamma^{-}_{e,h}(E). \quad \text{(S41)}$$

Without microwaves, the four principal peaks correspond to two ground state and two excited state tunneling peaks at $eV = \pm(\Delta + \epsilon)$ and $eV = \pm(\Delta - \epsilon)$, respectively. The derivations for all of these four peaks are very similar, so that in the following we derive the behavior for one peak, which can be easily extended to the other peaks.

Derivation for excited state tunneling electron peak

These peaks are located at bias voltages of $eV = -(\Delta - \epsilon) - k\hbar\omega_r$, i.e. at the bias voltage where $\Gamma_e^+(E)$ is resonant. We assume that $k_BT \ll \epsilon$, such that the Fermi function can be approximated by a step function. In order to observe this peak, the following conditions have to be fulfilled:

1. The tunneling rate $\Gamma_e^+(E)$ is resonant, i.e. $eV = -(\Delta - \epsilon) - k\hbar\omega_r$
2. The other tunneling rate $\Gamma_h(E)$ is nonzero, i.e. $|\epsilon + eV + l\hbar\omega_r| > \Delta$.
3. The difference in Fermi functions is nonzero, i.e. $|f(\epsilon - eV - k\hbar\omega_r) - f(\epsilon + eV + l\hbar\omega_r)| = 1$.

Combining the first condition with the other two conditions yields

$$k > \frac{2\epsilon}{\hbar\omega_r} + l \quad \text{(S42)}$$

Owing to the second condition, we approximate $\Gamma_h(E)$ by a constant, i.e. $\langle\Gamma_h\rangle = \Gamma_h(E \gg \Delta)$. Applying these conditions to Eq. (S40), we find for the excited state electron tunneling current

$$I_{ex,e}(V) = -\frac{e}{\hbar\eta_R}\sum_{l=-\infty}^{\infty}\sum_{k>\frac{2\epsilon}{\hbar\omega_r}+l} J_k^2(\alpha)\,J_l^2(\alpha)\,\Gamma_e^+(\epsilon - eV - k\hbar\omega_r)\langle\Gamma_h\rangle \quad \text{(S43)}$$

In analogy to the Tien-Gordon model, we define a bare tunneling current which does not involve the modulation by the microwaves $I^0_{ex,e}(V) = -\frac{e}{\eta_s\hbar}\langle\Gamma_h\rangle\Gamma_e^+(\epsilon - eV)$. Equation (S43) simplifies to

$$I_{ex,e}(V) = \sum_k w(\alpha,k)J_k^2(\alpha)I^0_{ex,e}(V + k\hbar\omega_r/e), \quad \text{(S44)}$$

where we have defined the weight function $w(\alpha,k)$ as

$$w(\alpha,k) = \sum_{m>m_0-k} J_m^2(\alpha) \quad \text{(S45)}$$

and where $m_0 = \lceil \frac{2\epsilon}{\hbar\omega} \rceil$, where $\lceil\rceil$ is the ceiling function ($\lceil x \rceil$ is defined as x rounded to the next larger integer). The weight function does not change the position nor the number of the replicas. It only modifies the amplitude of the peak. This nicely explains the appearance of replica at integer multiples of $\hbar\omega/e$ instead of $\hbar\omega/2e$. The weight function also introduces a threshold through the condition $m > m_0 - k$, which means that m_0 quanta of $\hbar\omega$ have to be absorbed from the microwave in order to excite the YSR state. The leading edge of the weight function determining the onset of the peak as function of microwave intensity is given by the lowest order Bessel function $J^2_{m_0-k}(\alpha)$. This means in particular that the bare tunneling current $I^0_{ex,e}(V)$ as defined above cannot be observed when the microwave is turned off.

Simplified tunneling equations for ground state and excited state tunneling

We can straightforwardly extend the above derivation for all four peaks. We find for the bare tunneling currents

$$I^0_{\mathrm{ex,e}}(V) = -\frac{e}{\eta_{\mathrm{R}}\hbar}\Gamma^+_e(\epsilon - eV)\langle\Gamma_h\rangle, \quad \text{(S46)}$$

$$I^0_{\mathrm{ex,h}}(V) = +\frac{e}{\eta_{\mathrm{R}}\hbar}\langle\Gamma_e\rangle\Gamma^+_h(\epsilon + eV), \quad \text{(S47)}$$

$$I^0_{\mathrm{gr,e}}(V) = +\frac{e}{\eta_{\mathrm{R}}\hbar}\Gamma^-_e(\epsilon - eV)\langle\Gamma_h\rangle, \quad \text{(S48)}$$

$$I^0_{\mathrm{gr,h}}(V) = -\frac{e}{\eta_{\mathrm{R}}\hbar}\langle\Gamma_e\rangle\Gamma^-_h(\epsilon + eV), \quad \text{(S49)}$$

where the first index (gr,ex) refers to ground state and excited state tunneling and the second index (e,h) refers to electron and hole tunneling, respectively. From these bare tunneling currents, which have one peak each, we find the following equations to calculate the spectra with microwaves

$$I_{\mathrm{ex,e}}(V) \approx \sum_k w(\alpha,k)J^2_k(\alpha)I^0_{\mathrm{ex,e}}(V + k\hbar\omega_{\mathrm{r}}/e), \quad \text{(S50)}$$

$$I_{\mathrm{ex,h}}(V) \approx \sum_k w(\alpha,k)J^2_k(\alpha)I^0_{\mathrm{ex,h}}(V - k\hbar\omega_{\mathrm{r}}/e), \quad \text{(S51)}$$

$$I_{\mathrm{gr,e}}(V) \approx \sum_k \tilde{w}(\alpha,k)J^2_k(\alpha)I^0_{\mathrm{gr,e}}(V - k\hbar\omega_{\mathrm{r}}/e), \quad \text{(S52)}$$

$$I_{\mathrm{gr,h}}(V) \approx \sum_k \tilde{w}(\alpha,k)J^2_k(\alpha)I^0_{\mathrm{gr,h}}(V + k\hbar\omega_{\mathrm{r}}/e), \quad \text{(S53)}$$

where the weight functions are defined as

$$w(\alpha,k) = \sum_{m\geq m_0-k} J^2_m(\alpha), \quad \text{(S54)}$$

$$\tilde{w}(\alpha,k) = \sum_{m\geq -m_0-k} J^2_m(\alpha). \quad \text{(S55)}$$

where $m_0 = \left\lceil \frac{2\epsilon}{\hbar\omega_{\mathrm{r}}} \right\rceil$ is the minimum number of quanta needed to excite the YSR state. Interestingly, we find that for ground state tunneling the weight function $\tilde{w}(\alpha,k)$ does not impose a threshold for the activation of the tunneling process, because the condition $m \geq -m_0$ (for $k = 0$) always includes the zeroth order Bessel function, such that resonant Andreev processes are always possible without microwaves as has been discussed before [11].

Quasiparticle tunneling from the ground state

For completeness, we note that quasiparticle tunneling has to be considered, when modeling ground state tunneling, since the lifetime of the YSR state is not infinite in practice. In the deep tunneling regime, quasiparticle tunneling can be calculated from the Tien-Gordon model

$$I_{\mathrm{qp}}(V,\alpha) = \sum_n J^2_n(\alpha)\, I(V + n\hbar\omega_{\mathrm{r}}/e, 0)\,. \quad \text{(S56)}$$

As Andreev processes become more dominant with increasing tunneling conductance, the quasiparticle current reduces (cf. also regularized quasiparticle current in Eq. (S33)) [1, 11]. Excited state tunneling is a two-electron tunneling process, so that quasiparticle tunneling does not apply for that process. The full Green's function model naturally includes all current contributions.

RESONANT VS. NONRESONANT ANDREEV PROCESSES

In Section , we have derived a simple model that finds a spacing of $\hbar\omega/e$ between the replica of the resonant Andreev processes despite two charges being transferred. By contrast, the replica of nonresonant Andreev reflections are spaced by $\hbar\omega/2e$. We can explain this difference in behavior by deriving a simplified equation for the regular Andreev reflection starting from the same Eq. (S32) as for the resonant Andreev processes. The main difference is that the anomalous Green's function $(g_{\mathrm{R}})_{12}(E)$ is no longer given by a resonance, but by the standard result

$$(g_{\mathrm{R}})_{12}(E) = \rho_{\mathrm{R}}\frac{\Delta_{\mathrm{R}}}{\sqrt{\Delta^2_{\mathrm{R}} - \omega^2}}, \quad \text{(S57)}$$

which in the following we will approximate by a constant, such that $|(g_{\mathrm{R}})_{12}(E)|^2 = \rho^2_{\mathrm{R}}$. This is justified because the relevant part of the anomalous Green's function that is probed here is very close to zero energy. We further define

$$\tilde{\Gamma}_{\mathrm{e}}(E) = \tilde{\Gamma}_{\mathrm{h}}(E) = 2\pi|t|^2\rho_{\mathrm{L}}(E)\rho_{\mathrm{R}}, \quad \text{(S58)}$$

Equation (S32) changes for nonresonant Andreev reflections into

$$I_{\mathrm{AR}}(V) = \frac{2e}{h}\sum_{k,l} J^2_k(\alpha)J^2_l(\alpha)\times\int_{-\infty}^{\infty}\tilde{\Gamma}_{\mathrm{e}}(E - eV - k\hbar\omega_{\mathrm{r}})\,\tilde{\Gamma}_{\mathrm{h}}(E + eV + l\hbar\omega_{\mathrm{r}})\,[f(E - eV - k\hbar\omega_{\mathrm{r}}) - f(E + eV + l\hbar\omega_{\mathrm{r}})]\,dE. \quad \text{(S59)}$$

For simplicity, we approximate the coherence peaks in $\rho_{\mathrm{L}}(E)$ by very sharp Lorentzians at $E = \pm\Delta$ with a very small width η_{L}, which can be easily integrated. The Fermi functions do not impose any restrictions here. We find

$$I_{\mathrm{AR}}(V) = \frac{\pi e}{h\eta_{\mathrm{L}}}\sum_{k,l} J^2_k(\alpha)J^2_l(\alpha)\frac{4\pi^2|t|^4\rho^2_{\mathrm{L}}\rho^2_{\mathrm{R}}}{(eV - \Delta + (k+l)\frac{\hbar\omega_{\mathrm{r}}}{2})^2 + \eta^2_{\mathrm{L}}}, \quad \text{(S60)}$$

which is again a Lorentzian that nicely shows how the replica are spaced by $\hbar\omega/2e$. This demonstrates that depending on the presence of a resonance inside the superconducting gap, the spacing between replica changes accordingly. In this case, the spacing between replica cannot be used for inferring the number of charges being transferred.

SUPPLEMENTARY REFERENCES

* Corresponding author; electronic address: c.ast@fkf.mpg.de

[1] H. Huang, C. Padurariu, J. Senkpiel, R. Drost, A. L. Yeyati, J. C. Cuevas, B. Kubala, J. Ankerhold, K. Kern, and C. R. Ast, *Tunnelling dynamics between superconducting bound states at the atomic limit,* Nature Physics **16**, 1227 (2020).

[2] S. Karan, H. Huang, C. Padurariu, B. Kubala, A. Theiler, A. M. Black-Schaffer, G. Morrás, A. L. Yeyati, J. C. Cuevas, J. Ankerhold, K. Kern, and C. R. Ast, *Superconducting quantum interference at the atomic scale,* Nature Physics **18**, 893 (2022).

[3] H. Huang, R. Drost, J. Senkpiel, C. Padurariu, B. Kubala, A. L. Yeyati, J. C. Cuevas, J. Ankerhold, K. Kern, and C. R. Ast, *Quantum phase transitions and the role of impurity-substrate hybridization in Yu-Shiba-Rusinov states,* Communications Physics **3**, 199 (2020).

[4] S. Karan, H. Huang, A. Ivanovic, C. Padurariu, B. Kubala, K. Kern, J. Ankerhold, and C. R. Ast, *Tracking a spin-polarized superconducting bound state across a quantum phase transition,* arXiv:2304.02955 (2023).

[5] H. Huang, S. Karan, C. Padurariu, B. Kubala, J. C. Cuevas, J. Ankerhold, K. Kern, and C. R. Ast, *Universal scaling of tunable Yu-Shiba-Rusinov states across the quantum phase transition,* Communications Physics **6**, 214 (2023).

[6] R. Drost, M. Uhl, P. Kot, J. Siebrecht, A. Schmid, J. Merkt, S. Wünsch, M. Siegel, O. Kieler, R. Kleiner, and C. R. Ast, *Combining electron spin resonance spectroscopy with scanning tunneling microscopy at high magnetic fields,* Review of Scientific Instruments **93**, 043705 (2022).

[7] J. C. Cuevas, J. Heurich, A. Martín-Rodero, A. Levy Yeyati, and G. Schön, *Subharmonic Shapiro Steps and Assisted Tunneling in Superconducting Point Contacts,* Phys. Rev. Lett. **88**, 157001 (2002).

[8] J. C. Cuevas, A. Martín-Rodero, and A. L. Yeyati, *Hamiltonian approach to the transport properties of superconducting quantum point contacts,* Phys. Rev. B **54**, 7366 (1996).

[9] A. Villas, R. L. Klees, H. Huang, C. R. Ast, G. Rastelli, W. Belzig, and J. C. Cuevas, *Interplay between yu-shiba-rusinov states and multiple andreev reflections,* Physical Review B **101**, 235445 (2020).

[10] S. A. González, L. Melischek, O. Peters, K. Flensberg, K. J. Franke, and F. von Oppen, *Photon-assisted resonant Andreev reflections: Yu-Shiba-Rusinov and Majorana states,* Phys. Rev. B **102**, 045413 (2020).

[11] M. Ruby, F. Pientka, Y. Peng, F. von Oppen, B. W. Heinrich, and K. J. Franke, *Tunneling processes into localized subgap states in superconductors,* Physical Review Letters **115**, 087001 (2015).
